# A Bibliometric Analysis of Research Trends in Geopolymer

**DOI:** 10.3390/ma15196979

**Published:** 2022-10-08

**Authors:** Jabulani Matsimbe, Megersa Dinka, David Olukanni, Innocent Musonda

**Affiliations:** 1 Department of Civil Engineering Science, Faculty of Engineering and the Built Environment, University of Johannesburg, Johannesburg 2006, South Africa; 2 Centre for Applied Research and Innovation in the Built Environment (CARINBE), Faculty of Engineering and the Built Environment, University of Johannesburg, Johannesburg 2092, South Africa; 3 Department of Mining Engineering, Malawi University of Business and Applied Sciences, P/Bag 303, Chichiri, Blantyre 3, Malawi; 4 Department of Civil Engineering, Covenant University, 10 Idiroko Road, Ota 112104, Ogun State, Nigeria

**Keywords:** geopolymer, geopolymer mortar, geopolymer concrete, inorganic polymers

## Abstract

Geopolymer is an inorganic material formed through the chemical reaction of an aluminosilicate precursor and an alkaline or acidic activating solution. It is seen as a green new alternative binder to ordinary Portland cement (OPC) for sustainable infrastructure development. The strength of the unary or blended geopolymer product is dependent on the composition and properties of the polymeric gel influenced by the ratios of Al_2_O_3_/SiO_2_, CaO/SiO_2_, CaO/(SiO_2_ + Al_2_O_3_), Na_2_SiO_3_/NaOH, SiO_2_/Na_2_O, and liquid/binder (L/B). Essential scientific inquiry has been progressively addressed by utilizing expert assessment and research metrics. The network visualization of bibliometric co-occurrence and co-citations is of particular significance. The present study aims to highlight the trends and progress of the most influential publication sources, keywords, authors, articles, and countries in geopolymer research in the last 10 years. Bibliometric data were retrieved through Scopus and visualized in VOSviewer to create bibliometric networks. The yearly distribution and growth trends (April 2011–2022) of geopolymer, geopolymer mortar, and geopolymer concrete before (after) applying inclusion criteria were from 754 to 9887 (5186), 47 to 1374 (866), and 145 to 3721 (2253), respectively, attributed to the discoveries in more precursor materials such as laterite and the growing interest in fire and heat-resistant structures, water and wastewater treatment, cement and concrete, and brick manufacturing. The top three journals in terms of prestige for geopolymer publications were the Journal of Hazardous Materials with an impact factor equal to 14.224 and h-index equal to 307, Cement and Concrete Research with an impact factor equal to 11.958 and h-index equal to 239, and the Journal of Cleaner Production with an impact factor equal to 11.072 and h-index equal to 232. The top three journals in terms of average citation per document were Cement and Concrete Research (135.75), Materials and Design (75), and Cement and Concrete Composites (68.35). Keywords such as “geopolymers”, “inorganic polymer”, “geopolymer”, “compressive strength”, “fly ash”, and “geopolymer concrete” had the highest occurrences in publications. John Provis—University of Sheffield, Prinya Chindaprasirt—Khon Kaen University, and Jay Sanjayan—Swinburne University of Technology had the highest total citations of 6377, 5626, and 4311, respectively. The highest number of publications were from China, India, Australia, the United States of America, and Malaysia. The bibliometric findings from this study can act as a tool for academicians and policymakers to exchange research expertise, collaborate on novel geopolymer research, and create innovative joint ventures.

## 1. Introduction

Geopolymer is an inorganic polymeric material synthesized by the reaction of an aluminosilicate powder with an alkaline solution through the mechanism of dissolution, gelation, and polycondensation that results in a hardened 3D polymeric ring and chain matrix of polysialate. The use of geopolymer is considered a new, better alternative binder [1,2] whose benefits compared to ordinary Portland cement consist of lower CO_2_ emissions [3], waste materials’ reutilization [4], supporting the circular economy solid waste management [5], higher thermal resistance [6], better mechanical performance [7], acid resistance [8], sulfate attack resistance [9], lower drying shrinkage [10], and freeze–thaw resistance [11]. The manufacture of ordinary Portland cement emits approximately 1350 million tons of greenhouse gases annually where the three most significant sources are fuel combustion (325 kg/ton CO_2_), limestone decarbonation (525 kg/ton CO_2_), and electrical energy consumption (50 kg/ton CO_2_) [12]. For similar mechanical properties, geopolymer offers a strong reduction in global warming by releasing 169 kg CO_2_/m^3^ compared to ordinary Portland cement, which releases 306 kg CO_2_/m^3^, representing an emission decrease of 45% [13,14]. Therefore, the production of ordinary Portland cement leads to massive CO_2_ emissions, consumes a lot of energy, and depletes natural resources. The adoption of green building materials for infrastructural development is strongly advocated by the International Energy Agency Global Roadmap for Buildings and Construction 2020–2050 [15,16]. The disposal of industrial waste materials in landfills causes tremendous environmental problems consisting of groundwater contamination, bulk storage spillage, and mobilization of heavy metals [17,18,19]. The utilization of these industrial byproducts and waste materials in geopolymer development improves the mechanical properties of concrete and reduces the environmental impact [20,21,22]. The polymerization approach effectively converts different industrial waste materials, e.g., fly ash, mine tailings, slag, phosphogypsum, red mud, etc., into value-added products such as binders, mortar, concrete, bricks, and foam. Approximately one billion tons of fly ash and 160 million tons of phosphogypsum are produced annually and only about 15–30% is utilized by industry [23]. The term “geopolymer” was coined in the 1970s by the French scientist and engineer (Prof Davidovits) and applied to a class of solid materials synthesized by the reaction of an aluminosilicate powder with an alkaline solution [2,24]. 

Geopolymerization refers to the chemical reaction process between an aluminosilicate source material and an alkaline activating solution producing a 3D polymeric ring and chain matrix of polysialate having an empirical formula Mn[−(SiO_2_)z − AlO_2_]n.wH_2_O, where M is a cation (K^+^, Na^+^, Ca_2_^+^), n is the polycondensation degree, and z is 1, 2, 3, or greater [2,24]. Sialate refers to a “silicon-oxo-aluminate” building unit whose network consists of SiO_4_ and AlO_4_ tetrahedra linked by sharing all oxygen atoms. The presence of the cations balances the remaining anions of the four coordinated Si^4+^ and Al^3+^ ions. The geopolymerization mechanism consists of dissolution, gelation, and polycondensation. Depending on the mix design, the final geopolymer matrix might consist of C–S–H, C–A–S–H, and/or N–A–S–H gels. The strength of the geopolymer product in unary or binary mixtures is dependent on the composition and properties of the polymeric gel influenced by the ratios of Al/Si, Ca/Si, Ca/(Si + Al), and SiO_2_/Na_2_O [25]. Several researchers have investigated the properties of geopolymer made from different precursor materials comprising fly ash [26], ground granulated blast furnace slag [27], bottom ash [28], mine tailings [29], rice husk ash [30], palm oil fuel ash [31], phosphogypsum [32], red mud [33], metakaolin [34], laterite [35], volcanic ash [36], and zeolite [37]. The major chemical compositions of the different precursor materials consist of Al_2_O_3_, SiO_2_, CaO, Fe_2_O_3_, and MgO, which chemically react with an alkaline activating solution under ambient or elevated temperature curing conditions to produce geopolymer products. Publications on geopolymer strive to provide an advanced understanding of the chemical, physical, and mechanical performance of the geopolymer binder, mortar, and concrete with respect to the mix design. However, the common factors limiting the extensive industrial application of geopolymer comprise the variable waste material properties [4], high curing temperature [38], high energy demand and cost of alkaline activators [39], caustic nature of alkaline and acidic activators [39], efflorescence [40], alkali-silica reaction [41], and the absence of mix design standards [42]. It is therefore imperative that readers are made aware of the research trends and directions in geopolymer technology.

Essential scientific inquiry has been progressively addressed by utilizing expert assessment and research metrics. To address the shortfalls of manual reviews in engineering studies and other disciplines [43], bibliometric analysis has provided a profound, quantitative, less skewed linkage and network between various aspects of large bibliometric data. The weaknesses of manual reviews consist of deficiencies in the development of an intense linkage and network between various aspects of large bibliometric data. Bibliometric analysis is a common and robust method for exploring and analyzing large volumes of scientific data [44]. Reference [45] used CiteSpace software to create bibliometric maps on geopolymer data retrieved from the Web of Science ranging from 1990 to 2017. They found that the majority of geopolymers are based on “fly ash” and “metakaolin” as aluminosilicate source materials and the most used leaching method is the “Toxicity Characteristic Leaching Procedure”. Reference [46] conducted a scientometric review of geopolymer concrete by analyzing 2011 journal articles/conference papers extracted from Scopus (March 2008–2020). The VOSviewer network visualization of keywords showed that “geopolymers and “inorganic polymers” have the highest frequencies of usage by researchers in the field. It was further observed that John Provis, Susan Bernal, Prinya Chindaprasirt, and Jannie van Deventer are the most influential authors in terms of citations and documents produced. Similar results on the top keywords and authors were found by [47] who conducted a scientometric evaluation of geopolymer composites data (geopolymers: 4950 articles and geopolymer concrete: 1826 articles) retrieved from Scopus (August 2011–2021) and analyzed in VOSviewer. Reference [48] conducted a bibliometric analysis of fly ash geopolymer by collecting a total of 4352 publications from the Web of Science (1998–2022) and visualizing the data in CiteSpace. They observed that the fly ash-based geopolymer field can be divided into three stages, namely, mechanical properties, the development of multifunctional ground polymer materials, and the synergistic use of different solid wastes to minimize environmental impact.

With the growing interest in geopolymer studies, there is a need to conduct a bibliometric analysis of the published literature to update readers and policymakers on current and future research hotspots. Despite the previous limited reviews and bibliometric studies, no bibliometric study has been conducted incorporating geopolymer in a broader sense of search strings providing wider bibliographic data for a potentially unbiased and less subjective representation of the trends and progress in the field. It is not clear which areas of geopolymer research are well developed and which are lacking. This study aims at highlighting the trends and progress of the most influential publication sources, keywords, authors, articles, and countries in geopolymer research in the last 10 years. The bibliometric analysis of the present study provides valuable statistical insights into geopolymer development and application in the industry, thereby showcasing the current and future research trends. It further provides a better understanding of the theoretical structure and cluster themes of geopolymer and identifies the important topics and significant contributions of top publications and authors in the evolution of the field. The network visualization of bibliometric co-occurrence and co-citations is of particular significance since it acts as a guide for academics and research institutions to exchange research expertise, share ingenious technologies, collaborate on novel research, and create innovative joint ventures. A bibliometric network is constructed and visualized establishing the yearly distribution and growth trend, leading publication sources, keyword co-occurrence, leading authors, top articles, and leading countries in geopolymer research.

## 2. Methodology

The present study adopted the bibliometric analysis technique to quantitatively visualize scientific data and create bibliometric maps. The technique is appropriate for this review since it highlights and analyzes the growth of geopolymer research over a period using large bibliographic data. Google Scholar, Scopus, and Web of Science are the most used citation indexing and academic literature collection databases [49,50]. Scopus has been the commonly preferred alternative to Web of Science by researchers due to its wider coverage of bibliometric data, broader journal range, and faster indexing process [49,50,51]. VOSviewer is a commonly used data visualization software developed by Nees Jan VanEck and Ludo Waltman of Leiden University in the Netherlands. It can create maps of authors, publications, and journals based on citation data, and keywords based on co-occurrence data [52]. Authors [7,12,46] retrieved their bibliometric data from Scopus and used VOSviewer to construct their bibliometric maps. Other researchers have utilized different bibliometric mapping software such as CiteSpace [17,48] developed by Professor Chaomei Chen of Drexel University in the USA, Bibliometrix [53] developed by Professor Massimo Aria of University of Naples Federico II in Italy, and Professor Corrado Cuccurullo of University of Campania Luigi Vanvitelli in Italy, Publish or Perish [54] developed by Professor Anne-Wil Harzing of Middlesex University in the UK, Pajek [55] developed by Professor Vladimir Batagelj and Professor Andrej Mrvar of University of Ljubljana in Slovenia, CitiNetExplorer [56] developed by Nees Jan VanEck and Ludo Waltman of Leiden University in the Netherlands, etc., depending on the type of analysis to be performed. Compared to VOSviewer, the other different software provide simple graphical representations and small bibliometric maps, which are not displayed in a satisfactory manner [52]. The present review aims to highlight the trends and progress in geopolymer research reflected through the most influential publication sources, keywords, authors, countries, and articles in the last 10 years containing moderately large bibliometric data. Therefore, this review used Scopus as a data retrieval tool and VOSviewer as a network mapping technique due to the large bibliometric data requiring more documents per download (>2000) and large graphical representation of bibliometric maps, respectively. An inclusion criterion was developed to filter out irrelevant journal articles for the present investigation. Table 1 shows the inclusion criteria applied to filter out unnecessary journal articles. 

The eligibility criteria, information sources, and search strategies were defined. The author’s search strings defined to conduct the research were “geopolymer”, “geopolymer mortar”, and “geopolymer concrete”. The abstract for each appeared document was read to ensure adherence to the author’s search strings. The data were retrieved in the format of a Comma Separated Values (CSV) file in April 2022, which was further imported and analyzed in VOSviewer software (version 1.6.18). Quality assurance on the imported data was performed in Microsoft Excel to search for and remove duplicates. A bibliometric network was constructed and visualized establishing the yearly distribution and growth trend, keyword co-occurrence, top publication sources, authors, articles, and countries in geopolymer research. 

## 3. Results and Discussion

This section presents the bibliometric analysis performed on the existing literature on geopolymer establishing the yearly distribution and growth trends, top publication sources, keyword co-occurrence, leading authors, top articles, and leading countries in geopolymer research.

### 3.1. Yearly Distribution and Growth Trends

Table 2 shows the documents returned for all search strings in Scopus as of 25 April 2022. For the “geopolymer” keyword, the first article was published in 1979, and this grew cumulatively to 754 articles up to 2010. Thereafter, there was an increase in geopolymer articles to 9887 from 2011 to 2022. Similarly, for the keyword “geopolymer mortar”, the first article was published in 2003 and this grew cumulatively to 47 articles up to 2010. Thereafter, there was an increase in geopolymer mortar articles to 1374 from 2011 to 2022. Lastly, for the keyword “geopolymer concrete”, the first article was published in 1989 and this grew cumulatively to 145 articles up to 2010. Thereafter, geopolymer concrete articles increased to 3721 from 2011 to 2022. 

**Figure 1:**
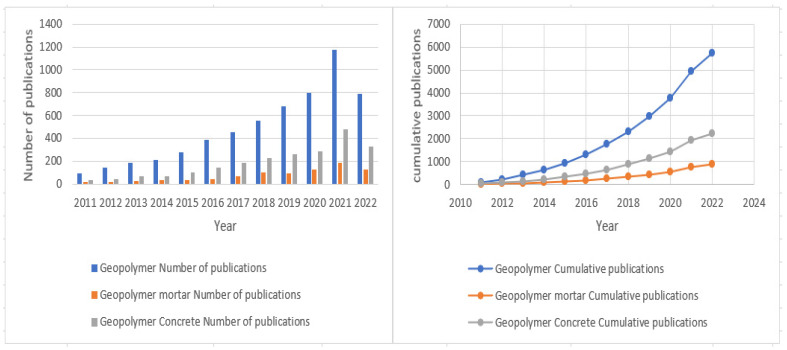
Year-on-year number of publications and cumulative publication trend for the searched keywords as of 25 April 2022.

Research interest in geopolymer has grown by more than 93% from 2011 to April 2022. An increase in the number of publications shows the growing interest in the geopolymer research field as a potential alternative to ordinary Portland cement for sustainable infrastructural development. The findings from the present study closely agree with [12] who divided their bibliometric review into three periods and observed that research in eco-friendly construction materials has gone through an emerging period (2001–2008), a pickup and pace period (2009–2015), and finally a rapid growth period (2016–2021). In another study, Reference [45] reported the bibliographic and visualized analysis of geopolymer research in three stages (1990–2003; 2004–2014; 2015–2017) and found that geopolymer publication outputs, respectively, increased slowly, gradually, and then rapidly in the last three years. Figure 1 shows the yearly publication trend for the searched keywords. Initially, a total of 14,982 articles (the sum of all keywords searched) were identified through Scopus. After applying the inclusion criteria, a total of 8305 articles were retained.

The quantity and analysis of publications on a time-period basis give a good estimate of the trends and status quo in a specific research field and can inform researchers of the probable future research trends in the area. The findings show an increase in geopolymer research, which can be attributed to the discoveries of more precursor materials such as laterite [35] and growing interest in applications such as fire and heat-resistant structures [30], water and wastewater treatment [57], immobilization of heavy metals [45], cements and concretes [58], and brick manufacturing [26]. Furthermore, researchers striving to optimize mix designs and strength, develop low-cost user-friendly activators, utilize low-temperature curing conditions, and advance durability properties have influenced the growth of geopolymer research. With the promotion and advancement of the circular economy in solid waste management [5,59,60], geopolymer research has had exponential growth due to the large availability of different industrial byproducts and/or waste materials such as rice husk ash, fly ash, bottom ash, waste glass, palm oil fuel ash, phosphogypsum, ground granulated blast furnace slag, red mud, etc. The increase in the yearly distribution and growth trend of geopolymer research, as a potential green building material, can also be attributed to the Sustainable Development Goals [61], the International Energy Agency GlobalABC Roadmap for Buildings and Construction 2020–2050 [15], and the UN Climate Change Conference of the Parties Paris Agreement [16].

### 3.2. Publication Sources Contribution

To evaluate growth in research output, publication sources mapping is essential. The top publication sources/journals with a minimum of 50 documents from April 2011 to April 2022 are shown in Table 3. Construction and Building Materials, the Journal of Cleaner Production, Ceramics International, and Materials are the top four most preferred sources for geopolymer publication having 1030, 239, 232, and 230 documents, respectively. These documents were able to garner 40,671, 7611, 7388, and 3240 citations, respectively. The top three sources in terms of average citation per document of 135.75, 75, and 68.35 were Cement and Concrete Research, Materials and Design, and Cement and Concrete Composites, respectively. 

**Figure 2:**
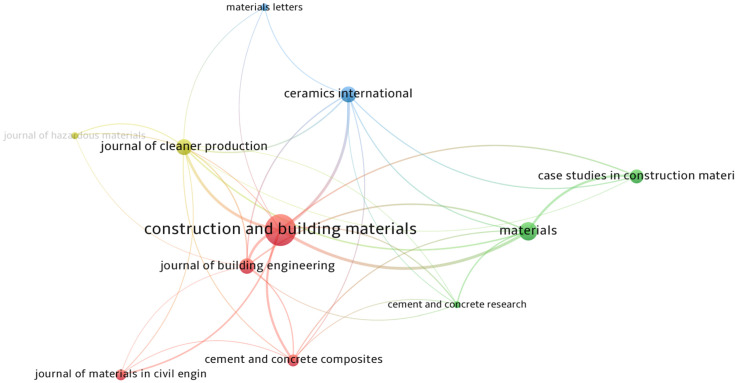
Visualization of publication sources with a minimum of 50 documents.

The journal impact factor (JIF) and h-index, shown in Table 3, were used to measure the importance of journals to the scientific community. A similar approach was used by [54,62,63] who mentioned that the h-index and JIF indicate the authority and influence of a journal. Therefore, the top three journals in terms of importance for geopolymer publications were the Journal of Hazardous Materials with a JIF equal to 14.224 and an h-index equal to 307, Cement and Concrete Research with a JIF equal to 11.958 and an h-index equal to 239, and the Journal of Cleaner Production with a JIF equal to 11.072 and an h-index equal to 232. The interest in these journals can be attributed to compliance with the Sustainable Development Goals [61], the International Energy Agency GlobalABC Roadmap for Buildings and Construction 2020–2050 [15], and the UN Climate Change Conference of the Parties Paris Agreement [16], which promote the use of non-hazardous materials [64], eco-friendly cement and concrete [65], and cleaner production [22] to address the growing issues in solid waste materials disposal and CO_2_ emission brought about by the increase in urbanization and population growth. Figure 2 shows the network visualization of the most preferred publication sources for geopolymer. The size of the circle indicates the journal’s publication count, i.e., the bigger the circle size the higher the number of documents published, but this does not indicate the authority/prestige of the journal. For example, the Construction and Building Materials journal has the biggest circle size and thus the highest number of published documents, but a lower JIF and h-index compared to the Journal of Hazardous Materials, Cement and Concrete Research, and the Journal of Cleaner Production. 

Furthermore, circles with a similar shade imply the publication sources’ association or interrelationship. The four distinct colors detected indicate four associated groups ranging from red (Group 1), green (Group 2), blue (Group 3), and yellow (Group 4) with 4, 3, 2, and 2 items, respectively. Publication sources with a similar color close together show a closer association compared to those far apart. Most of the publication sources are citing articles from Construction and Building Materials, Cement and Concrete Composites, Cement and Concrete Research, and the Journal of Cleaner Production. Ahmad et al. [12] reported the direct link between the top two publication sources comprising the Journal of Cleaner Production and Construction and Building Materials. Closely located publication sources have a similar cluster and the network edges indicate the linkages in citation [66].

### 3.3. Keyword Co-Occurrence

Keywords indicate the aim of the research and the subject focus of the document. They help researchers to understand the core content of a document and subject categories and identify the progress and trend of research in the field [67]. Table 4 highlights the most used keywords in geopolymer publications. Geopolymers, inorganic polymer, and geopolymer are the top three keywords with 4082, 4045, and 3067 occurrences, respectively. 

**Figure 3:**
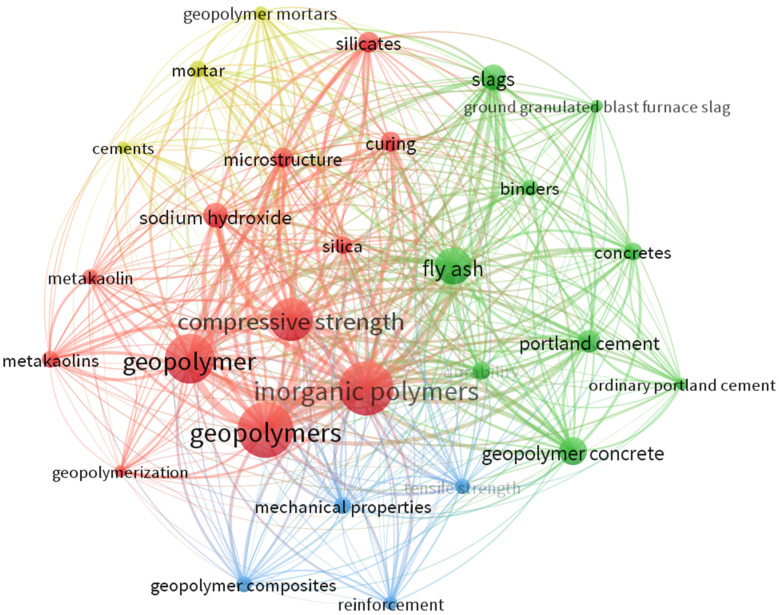
Network visualization of keyword co-occurrence.

Figure 3 shows the keyword co-occurrence network visualization where the size of the circle depicts the keyword usage frequency in the most important topics, and its location implies a correlation in publications. VOSviewer (Figure 3) detected four groups where red (Group 1), green (Group 2), blue (Group 3), and yellow (Group 4) had 12, 8, 4, and 3 keywords, respectively. It should be observed that keywords such as “Scanning electron microscope”, “Fourier transform infrared spectroscopy”, “Bins”, and “Blast furnaces” were omitted as they do not refer to any specific sub-area in geopolymer research. Keywords represent the content of a research domain and capture the core area of the specific research field [46].

It can be observed that keywords such as “geopolymers”, “inorganic polymer”, “geopolymer”, “compressive strength”, “fly ash”, and “geopolymer concrete” have higher occurrences and bigger circle sizes than the other keywords. This can be attributed to the growing research interest in optimizing mix design and strength as well as modeling the geopolymeric gel mechanism compared to Portland cement. Fly ash, silica, slag, and metakaolin are the commonly used precursors in unary or blended geopolymer systems either at elevated or ambient curing temperatures. Similar findings on fly ash and metakaolin as common precursors were observed by [45]. Most of these waste material precursors are usually blended with natural materials such as “metakaolin” to improve the mechanical properties of the base material [68]. However, “fly ash” has been the most used aluminosilicate source due to its pozzolanic and cementitious behavior, better physico-chemical properties, and availability [69,70] compared to the other industrial byproducts. The keywords “silicates”, “sodium hydroxide”, and “curing” can be attributed to the promotion of low-cost user-friendly activators [5], dosage optimization of sodium silicate plus sodium hydroxide activators [71], and growth in low curing temperatures [72] for geopolymer production. The keyword “reinforcement” is attributed to the growth in fiber-reinforced geopolymers [73,74,75] to improve the “mechanical properties”, “tensile strength”, and “durability”. The keyword “microstructure” is analyzed using X-ray diffraction (XRD), scanning electron microscopy (SEM), and nuclear magnetic resonance (NMR) spectroscopy [76] to understand the mineralogy, morphology, and molecular configuration of geopolymer, respectively. The keywords “binder”, “cement”, “concrete”, and “mortar” correspond to the applications of geopolymer. Therefore, this study’s findings can guide future researchers to choose keywords in active research areas and easily identify a particular subject in search engines.

### 3.4. Authors’ Contribution

The analysis of the author’s and co-author’s relationship provides significant information on the major research groups working in a certain discipline. The influence of a researcher in a specific field is assessed by the number of citations to their publications [56]. Therefore, the number of publications and citations shown in Table 5, measures the contribution and impact of a researcher in a particular field. The average citation is determined by dividing the total citations by the total number of documents per author. Jay Sanjayan, Prinya Chindaprasirt, and Mohd Mustafa Al Bakri Abdullah have the most published documents of 104, 99, and 91, respectively, whilst John Provis—University of Sheffield, Prinya Chindaprasirt, and Jay Sanjayan have the highest total citations of 6377, 5626, and 4311, respectively. In terms of average citations, the leading authors are John Provis, Vanchai Sata—Khon Kaen University, and Zuhua Zhang with around 133, 79, and 64 average citations, respectively. The analysis shows that researchers from different geographical areas are interconnected through citations in the field of geopolymer.

**Figure 4:**
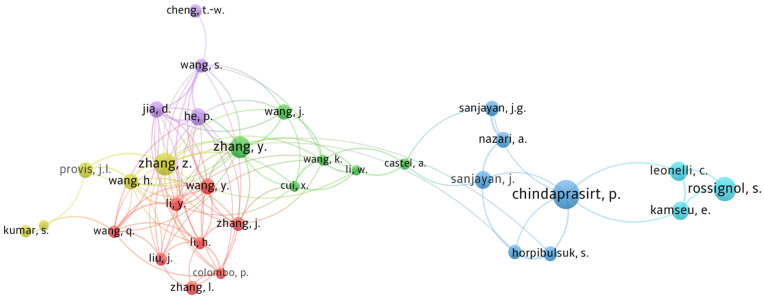
Visualization of authors’ contribution based on citations and co-authorship.

As shown in Figure 4, influence is achieved by mapping the author’s and co-author’s relationship. The mapping process provides a network visualization of an author’s activity and interconnectivity with others in the research field. Figure 4 shows that there are six major groups (considering at least three authors in a group) working in the field of geopolymer technology. The prominent research groups in the geopolymer research field are from the authors Sylvie Rossignol—Institut de Recherche sur les Céramiques, Prinya Chindaprasirt—Khon Kaen University, Jay Sanjayan—Swinburne University of Technology, Paolo Colombo—University of Padova, Zuhua Zhang—Hunan University, and Peigang He—Harbin Institute of Technology. Amongst all the researchers, Jay Sanjayan has the highest number of publications followed by Prinya Chindaprasirt, and Mohd Mustafa Al Bakri Abdullah—Universiti Malaysia Perlis, who have 104, 99, and 91 documents, respectively. The research groups of Paolo Colombo, Zuhua Zhang, and Peigang He were highly interconnected and together formed the largest cluster of 16 researchers. It is possible that Qingyuan Wang—Sichuan University and Dechang Jia—Harbin Institute of Technology were trained/affiliated with Colombo’s research laboratory before joining their current research laboratory. Invariantly, the research groups of Colombo, Zuhua Zhang, and Peigang He share their research expertise. 

### 3.5. Publication Contribution

The article and citation relationship informs researchers on how familiar the published article is. An article with a higher citation metric implies that the article quality might be excellent and therefore cited by many authors in the research field. The present study selected publications with a minimum of 400 citations, narrowing down the number of documents to 16 (Table 6). This assisted in the analysis of the highly cited publications and the most considerable publications having a significant impact on geopolymer research. 

With reference to Table 6, the top four most cited publications were “Costs and carbon emissions for geopolymer pastes in comparison to ordinary portland cement” by McLellan et al. [3], “Carbon dioxide equivalent (CO_2_-e) emissions: A comparison between geopolymer and OPC cement concrete” by Turner and Collins [77], “An environmental evaluation of geopolymer based concrete production: reviewing current research trends” by Habert et al. [14], and “Geopolymer concrete: A review of some recent developments” by Singh [78], which, respectively, had 884, 878, 675, and 649 citations. The increase in the citations shows the relevance of the publications in fostering geopolymer research. It can be noticed that the cited documents were comparing geopolymer to Portland cement based on the environmental impact, most probably to justify its use as a sustainable cement and assess its viability. However, the sustainability aspect is still debatable due to the CO_2_ emissions arising from the production of alkaline activators, the caustic nature of alkaline or acidic activators, and the high curing temperatures. This might be the reason why researchers have cited the documents (with reference to Table 6) by Nath and Sarker [79], and Deb et al. [83] since the attention now is on producing geopolymer products at ambient/low curing temperatures with low-cost user-friendly activators. Therefore, there is a need for more research to address the activator and curing challenges.

**Figure 5:**
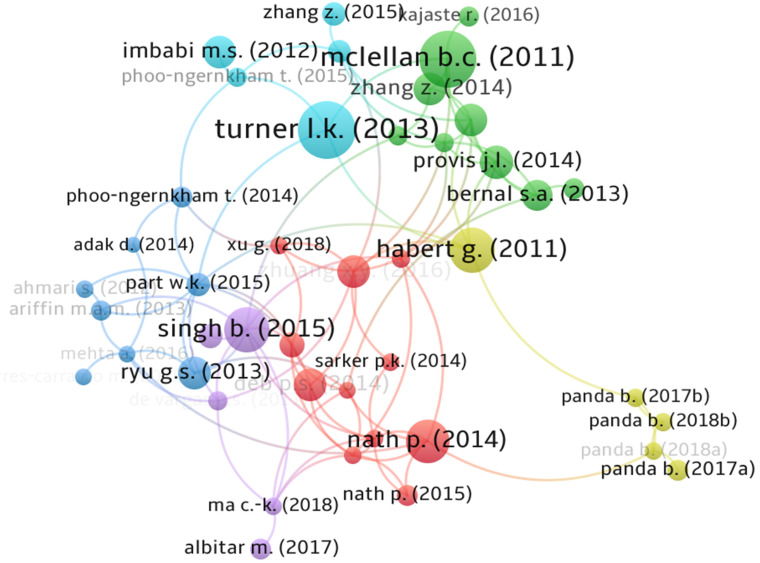
Visualization of publications with a minimum of 400 citations.

Figure 5 shows how the publications with a minimum of 400 citations are interlinked with others having at least 200 citations. VOSviewer (Figure 5) detected six groups where red (Group 1), green (Group 2), blue (Group 3), yellow (Group 4), purple (Group 5), and turquoise (Group 6) had 11, 9, 8, 5, 5, and 5 documents. The analysis shows that researchers from different geographical areas are interconnected through citations in the field of geopolymer. The network circle sizes for the documents [3,14,77,78,79] are larger compared to the others, implying their impact on geopolymer research across different geographical regions.

### 3.6. Countries’ Contribution

Awareness of the most active countries in a specific research field fosters future collaboration, promotes technology exchange, and facilitates joint venture funding [66]. Table 7 shows that a total of 6980 documents were published from 33 countries. The highest number of publications (1124 documents, 16.10% of the total documents) was from China, followed by 890 publications (12.75%) from India. Australia was ranked third with 651 publications (9.33%) whilst the United States of America ranked fourth with 451 publications (6.46%). Subsequently, seven countries published documents in the range of 200 to 350, nine countries in the range of 100 to 199, and thirteen countries in the range of 50 to 99. Furthermore, Table 7 depicts that out of the 33 countries, 14 countries had a nominal GDP rank of less than/equal to 15. This indicates that economically developed countries and India (a developing country) have identified the benefits of geopolymer research and are exploring their feasibility for construction applications. China has received the greatest number of citations (24,008) followed by Australia (21,231), and the United States of America (11,609). Interestingly, Finland and Singapore have the highest average number of citations per document (62.11) and (60.88), respectively, as compared to the other countries.

**Figure 6:**
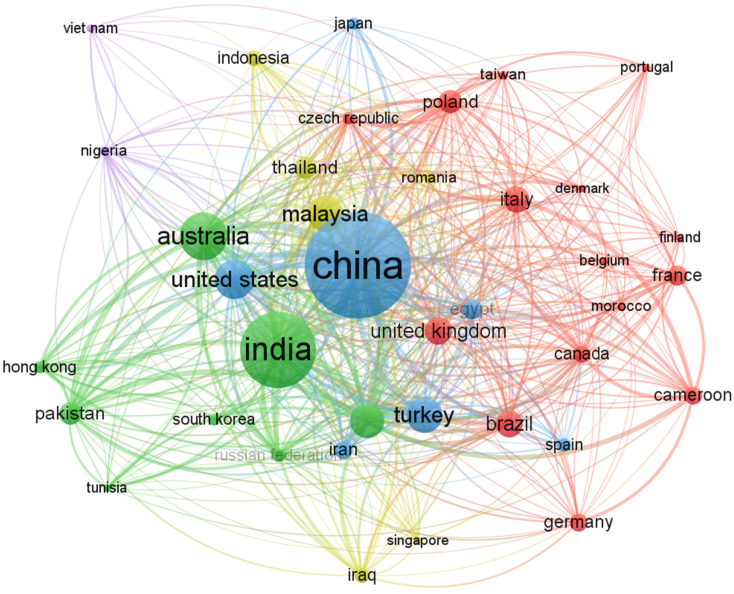
Visualization of countries with a minimum of 50 publications.

The country cooperation network (Figure 6) shows that there is a robust research collaboration between most of the countries with India, Australia, and China. To show commitment to the commercial implementation of geopolymer in Australia [91], fly ash-based geopolymer concrete was successfully utilized in the construction of the University of Queensland Global Change Institute Building in Australia, where 33 no. 11 m span precast floor beams made of geopolymer concrete (about 320 m^3^) formed three suspended floors in the building. Additionally, the Brisbane West Wellcamp Airport used about 40,000 m^3^ (100,000 tons) of geopolymer concrete and saved 6600 tons of CO_2_ emissions. The only African countries leading in geopolymer research consist of Egypt, Cameroon, Nigeria, and Morocco; hence the need to scale up geopolymer research in sub-Saharan Africa for sustainable infrastructural development. In South Africa, fly ash–slag geopolymer concrete was used to construct a concrete slab at the City Deep container terminal achieving a compressive strength of 51 MPa in 28 days [92]. Compared to many other regions in the world, the technologies of cement and concrete production are of great importance and relevance in the African context, making it the target destination for most large economies [93]. 

## 4. Future Research Trends

The directions for further research have been observed in the following areas:

- A synchronized standard geopolymer mix design and test method incorporating various ranges of NaOH, Na_2_SiO_3_/NaOH, SiO_2_/Na_2_O, SiO_2_/Al_2_O_3_, CaO/SiO_2_, CaO/(SiO_2_ + Al_2_O_3_), L/B, curing temperature and time, and aggregates to achieve better geopolymerization and give better strength output.
- The harmonized utilization of the different waste materials to obtain geopolymers with high performance. The properties of the waste materials vary making it difficult to develop geopolymers with consistent properties.
- Predictive models for mechanical strength and durability properties of geopolymer to guide preliminary mix design and achieve the required performance without conducting tedious and costly trial and error mix formulations.
- Techniques to enhance the reactivity of precursor materials.
- Low-temperature curing conditions to replace the high-temperature curing conditions, save on energy costs, and adopt in situ casting of geopolymer.
- Low-cost user-friendly activating solutions to replace the expensive user-hostile alkaline and acidic solutions.
- Large-scale treatment technology for phosphogypsum and its reaction mechanism in unary or binary geopolymer systems.
- The application and implementation of geopolymer binder, mortar, concrete, brick, etc., in the architecture, engineering, and construction (AEC) industry.

## 5. Limitations

Despite its contribution, this study has limitations. The dataset used for the bibliometric analysis is retrieved from the Scopus citation indexing database prone to the intrinsic and extrinsic coverage of publications. The literature retrieved is dependent on the searched keywords, which do not guarantee retrieval of alternate keywords adopted by other researchers such as “green cement”, “geocement”, etc. Non-English documents are excluded, which are likely to increase the retrieved information if included. This investigation is limited to journal articles and reviews. The observed limitations imply that the findings might not fully reflect all the available publications in databases dealing with geopolymer. Moreover, the utilization of citation networks as a measure of quality and impact may be prone to criticism. These limitations could serve as further research areas for bibliometric analysis on similar studies through the comprehensive inclusion of all document types and languages, and the comparative utilization of several different databases and bibliometric mapping software.

## 6. Conclusions

This study conducted the bibliometric analysis of geopolymer publications sourced from the Scopus database ranging from April 2011 to April 2022. Research publications are viewed as the “acceptable currency of scientific work” [46] that shows the advancement of science, archives groundbreaking outputs, and provides a forum for scientific theories/debates. References. [94,95] concluded that research metrics can assist researchers, governments, and industry to envision future innovative areas, prioritize funding areas, and assess the impact of past joint ventures. The present study observed that research on geopolymers is rapidly growing and that further research is needed to address gaps in large-scale civil engineering application and implementation. The key findings from the bibliometric analysis are summarized below:

- In terms of publication sources, the journals Construction and Building Materials, Journal of Cleaner Production, Ceramics International, and Materials are the top four most preferred for geopolymer publications. Most of the sources were citing articles from the journals Construction and Building Materials, Cement and Concrete Composites, Cement and Concrete Research, and the Journal of Cleaner Production.
- In terms of keyword co-occurrence, the most used keywords are geopolymers, inorganic polymer, and geopolymer. The findings can assist future researchers to choose keywords for easy identification of a particular research field in search engines.
- In terms of author contribution, the authors Jay Sanjayan, Prinya Chindaprasirt, and Mustafa Al Bakri Abdullah have the most documents, whilst John Provis has the most total citations. The analysis shows that researchers from different geographical areas are interconnected through citations in the field of geopolymer.
- In terms of publication contribution, the top three most cited publications were References. [3,14,77]. The network circle sizes for these documents are larger compared to the others, implying their impact on geopolymer research across different geographical regions.
- In terms of countries’ contribution, the highest number of publications were from China, India, Australia, and the United States of America. The country cooperation network depicts that there is a robust research collaboration of most of the countries with India, Australia, and China. The African countries leading in geopolymer research consist of Egypt, Cameroon, Nigeria, and Morocco; hence the need to scale up geopolymer research in sub-Saharan Africa.

## Figures and Tables

**Table 1:** Inclusion criteria applied when retrieving data from Scopus as of 25 April 2022.

Option	Inclusion Criteria Applied
Language	English
Publication date	April 2011–2022
Subject area	Engineering; Material Science; Environmental Science
Source type	Journal
Document type	Article, Review

**Table 2:** Searched keywords and returned documents from Scopus as of 25 April 2022.

S/N	Keywords Searched	Article Results	Article Results after Limits
**1**	Geopolymer	9887	5186
**2**	Geopolymer mortar	1374	866
**3**	Geopolymer concrete	3721	2253

**Table 3:** Publication sources with a minimum of 50 documents from April 2011 to April 2022.

S/N	Source	Publications	Citation	Average Citation per Publication	JIF	H-Index
**1**	Construction and Building Materials	1030	40,671	39.49	7.693	198
**2**	Journal of Cleaner Production	239	7611	31.85	11.072	232
**3**	Ceramics International	232	7388	31.84	5.532	126
**4**	Materials	230	3240	14.09	3.748	128
**5**	Cement and Concrete Composites	163	11,141	68.35	9.93	174
**6**	Journal of Materials in Civil Engineering	136	3518	25.87	3.266	114
**7**	Journal of Building Engineering	124	2077	16.75	5.318	54
**8**	Materials Letters	82	3567	43.50	3.574	155
**9**	Case Studies in Construction Materials	81	806	9.95	4.934	36
**10**	Cement and Concrete Research	73	9910	135.75	11.958	239
**11**	Journal of the American Ceramic Society	67	2859	42.67	3.784	203
**12**	Sustainability	60	342	5.70	3.889	109
**13**	Journal of Hazardous Materials	59	1328	22.51	14.224	307
**14**	Composites Part B Engineering	56	3412	60.93	9.078	163
**15**	Polymers	53	285	5.38	4.967	89
**16**	Materials and Design	52	3900	75.00	9.417	187

**Table 4:** Top occurring keywords in publications.

S/N	Keyword	Occurrences	S/N	Keyword	Occurrences
**1**	Geopolymers	4082	15	Binders	546
**2**	Inorganic Polymer	4045	16	Silica	529
**3**	Geopolymer	3067	17	Mortar	518
**4**	Compressive Strength	2587	18	Metakaolins	484
**5**	Fly Ash	2211	19	Polymer	463
**6**	Geopolymer Concrete	1102	20	Metakaolin	457
**7**	Slags	989	21	Cement	433
**8**	Silicates	877	22	Durability	427
**9**	Portland Cement	857	23	Tensile Strength	375
**10**	Sodium Hydroxide	827	24	Geopolymer Composites	368
**11**	Curing	813	25	Reinforcement	352
**12**	Concretes	774	26	Geopolymerization	343
**13**	Microstructure	695	27	Binders	546
**14**	Mechanical Properties	653	28	Silica	529

**Table 5:** Authors with a minimum of 30 publications.

S/N	Author	Publications	Citations	Average Citation	Total Link Strength
**1**	Jay Sanjayan	104	4311	41.45	182
**2**	Prinya Chindaprasirt	99	5626	56.83	181
**3**	Mohd Mustafa Al Bakri Abdullah	91	1010	11.10	377
**4**	Sylvie Rossignol	80	1778	22.23	239
**5**	Cristina Leonelli	61	1579	25.89	220
**6**	Elie Kamseu	59	1236	20.95	228
**7**	Zuhua Zhang	53	3376	63.70	246
**8**	Peigang He	50	1130	22.60	338
**9**	Dechang Jia	50	1119	22.38	332
**10**	Ali Nazari	48	1708	35.58	67
**11**	John Provis	48	6377	132.85	106
**12**	Suksun Horpibulsuk	44	2458	55.86	112
**13**	Xue-min Cui	44	1549	35.20	147
**14**	Arul Arulrajah	43	2009	46.72	107
**15**	Ta-Wui Cheng	39	797	20.44	97
**16**	Yingwu Zhou	39	924	23.69	283
**17**	Joao Labrincha	38	1559	41.03	132
**18**	Andrei Victor Sandu	38	565	14.87	168
**19**	Faiz Ahmed Shaikh	37	2238	60.49	63
**20**	Vanchai Sata	36	2829	78.58	85
**21**	Arnaud Castel	33	1389	42.09	30
**22**	Claudio Ferone	33	1202	36.42	121
**23**	Jian-Guo Dai	32	624	19.50	53
**24**	Xiafeng Duan	32	579	18.09	246
**25**	Yan He	32	927	28.97	83
**25**	Paolo Colombo	31	1083	34.94	78

**Table 6:** Documents with a minimum of 400 citations.

S/N	Publication	Citations
**1**	McLellan et al. [3]	884
**2**	Turner & Collins [77]	878
**3**	Habert et al. [14]	675
**4**	Singh et al. [78]	649
**5**	Nath & Sarker [79]	626
**6**	Ismail et al. [80]	537
**7**	Luukkonen et al. [81]	462
**8**	Provis, [82]	455
**9**	Deb et al. [83]	445
**10**	van Deventer et al. [84]	445
**11**	Zhang et al. [85]	439
**12**	Ryu et al. [86]	437
**13**	Imbabi et al. [87]	435
**14**	Zhuang et al. [88]	419
**15**	Bernal et al. [89]	411
**16**	He et al. [90]	406

**Table 7:** Countries with a minimum of 50 publications.

S/N	Country	Publications	Percentage (%)	Citations	Average Citation per Publication	Nominal GDP Rank (IMF 2022)
**1**	China	1124	16.10	24,008	21.36	2
**2**	India	890	12.75	11,234	12.62	5
**3**	Australia	651	9.33	21,231	32.61	13
**4**	United States	451	6.46	11,609	25.74	1
**5**	Malaysia	333	4.77	8777	26.36	34
**6**	Italy	299	4.28	7903	26.43	9
**7**	UK	238	3.41	5971	25.09	6
**8**	Thailand	234	3.35	7515	32.12	28
**9**	Turkey	215	3.08	3746	17.42	23
**10**	France	212	3.04	2936	13.85	7
**11**	Saudi Arabia	201	2.88	3209	15.97	18
**12**	Brazil	181	2.59	3748	20.71	10
**13**	Germany	153	2.19	4131	27.00	4
**14**	Iran	153	2.19	2707	17.69	14
**15**	Spain	144	2.06	3550	24.65	15
**16**	Egypt	141	2.02	2428	17.22	35
**17**	South Korea	124	1.78	1736	14.00	12
**18**	Cameroon	118	1.69	2644	22.41	95
**19**	Canada	115	1.65	3105	27.00	8
**20**	Czech Republic	100	1.43	681	6.81	48
**21**	Pakistan	97	1.39	877	9.04	44
**22**	Indonesia	96	1.38	588	6.13	17
**23**	Portugal	95	1.36	1113	11.72	51
**24**	Iraq	88	1.26	1339	15.22	47
**25**	Taiwan	80	1.15	1177	14.71	21
**26**	Romania	72	1.03	784	10.89	49
**27**	Hong Kong	58	0.83	1283	22.12	42
**28**	Finland	55	0.79	3416	62.11	46
**29**	Russia	55	0.79	321	5.84	11
**30**	Viet Nam	53	0.76	1350	25.47	39
**31**	Belgium	52	0.74	563	10.83	25
**32**	Nigeria	52	0.74	692	13.31	31
**33**	Singapore	50	0.72	3044	60.88	37

## Data Availability

Not applicable.
